# Crystal structure of aqua­(perchlorato)bis­[μ-(*E*)-2-({[2-(pyridin-2-yl)eth­yl]imino}­meth­yl)phenolato-κ^4^ *N*,*N*′,*O*:*O*]dicopper(II) perchlorate

**DOI:** 10.1107/S2056989017014694

**Published:** 2017-10-20

**Authors:** Ugochukwu Okeke, Yilma Gultneh, Ray J. Butcher

**Affiliations:** a Department of Chemistry, Howard University, 525 College Street NW, Washington, DC 20059, USA

**Keywords:** crystal structure, dinuclear Cu complex, coordinated water, coordinated perchlorate anions, Schiff base ligand complexes

## Abstract

The title compound, [Cu_2_(ClO_4_)(C_14_H_13_N_2_O)_2_(H_2_O)]ClO_4_, crystallizes as an unsymmetrical dinuclear cation bridged by the phen­oxy O atoms with one Cu^II^ atom coordinated by a water mol­ecule and the other by a perchlorate anion, thus making both Cu^II^ atoms five-coordinate, and with a further perchlorate anion present for charge balance. A long inter­action [2.9893 (5) Å] between one of the two Cu^II^ atoms and an O atom of the perchlorate counter-ion links the cations and anions into linear chains along the *a-*axis direction. In addition, the water H atoms link with the perchlorate counter-ion. These inter­actions, along with numerous C—H⋯O inter­actions between the tetra­hedral perchlorate anions, link the ions into a complex three-dimensional array. One of the perchlorate anions is disordered over two conformations with occupancies of 0.586 (4) and 0.414 (4).

## Abstract 2

The title compound crystallizes as an unsymmetrical dinuclear cation bridged by the phen­oxy O atoms with one Cu atom coordinated by a water mol­ecule and the other by a perchlorate anion, thus making both Cu atoms five-coordinate, and with a further perchlorate anion present for charge balance.

## Chemical context   

Proteins containing dinuclear copper centers play important roles in biology, including di­oxy­gen transport or activation, electron transfer, reduction of nitro­gen oxides and hydrolytic chemistry (Karlin & Tyeklar, 1993 ▸; Torelli *et al.*, 2000 ▸; Poater *et al.*, 2008 ▸; Utz *et al.*, 2003 ▸). The catalytic properties of some dicopper complexes have also been observed in some recent studies (Jagoda *et al.*, 2005 ▸). The crystal engineering of self-assembled supra­molecular architectures is currently of great inter­est, owing to their intriguing topologies and their applications in materials chemistry, in particular in optoelectronics, conductivity and superconductivity, charge-transfer and magnetism, nanoporous materials and biomimetic materials (Robson, 1996 ▸; Blake *et al.*, 1999 ▸; Sauvage, 1999 ▸).

Compounds of transition metal complexes comprising the ({[2-(pyridin-2-yl)eth­yl]imino}­meth­yl)phenol ligand have been synthesized for various processes (Egekenze *et al.*, 2017 ▸; Sanyal *et al.*, 2014 ▸; Chakraborty *et al.*, 2013 ▸; Tandon *et al.*, 1994 ▸, 2000 ▸; Latour *et al.*, 1989 ▸). Complexes of the tridentate ligand have been used as biomimics in the catalysis of hydrolysis of phosphate esters and as catalysts for catechol oxidation (Egekenze *et al.*, 2017 ▸). Pyrazole and pyridine are nitro­gen donors that are commonly used as ligands to mimic metalloenzymes. These heterocyclic groups are widely used to form inorganic complexes because they have pK_a_ values similar to those present in the hystidyl functional group of many enzymes. As part of an ongoing effort to synthesize complexes to use as biomimetics, the title copper(II) complex has been synthesized. In view of the inter­est in these types of metal complexes, its structure has been determined.
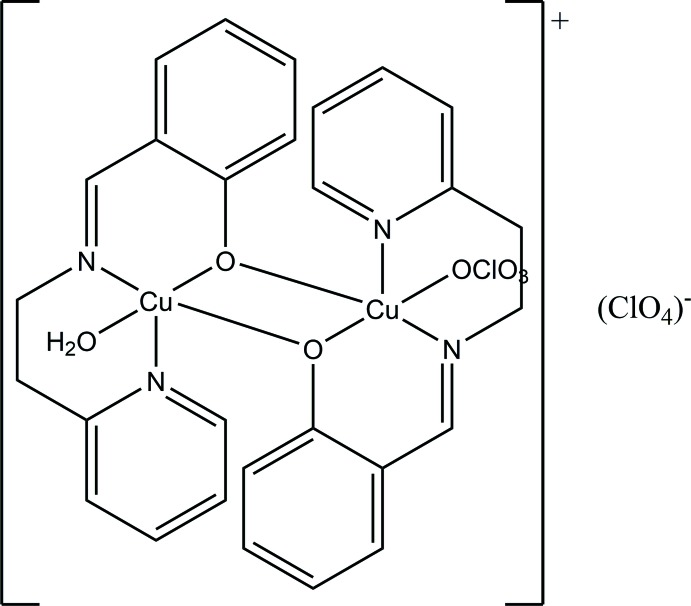


## Structural commentary   

**Figure 1:**
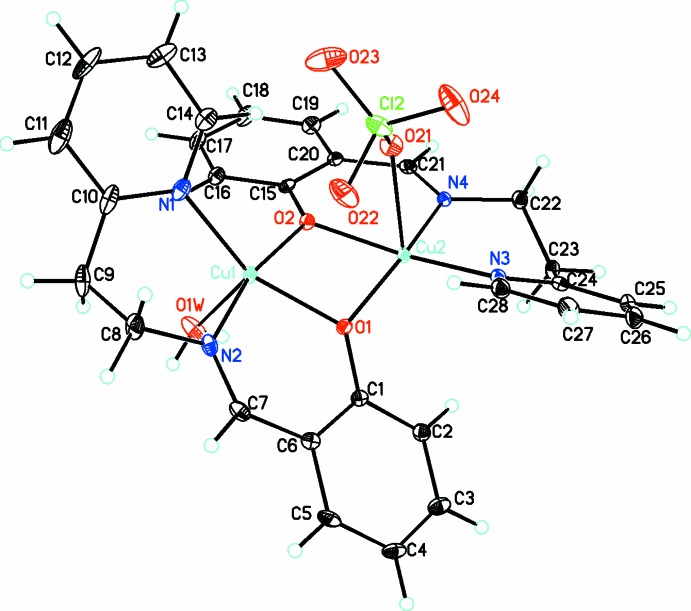
Diagram of the Cu-containing dinuclear cation showing the atom labeling. The non-coordinated anion is omitted for clarity. Displacement parameters are at drawn the 30% probability level.

The title compound crystallizes in the monoclinic space group *P*2_1_/*c* as an unsymmetrical dinuclear cation bridged by the phen­oxy O atoms with one Cu^II^ atom coordinated by a water mol­ecule and the other by a perchlorate anion, thus making both Cu^II^ atoms five-coordinate, and with a further perchlor­ate anion present for charge balance (see Fig. 1 ▸). The Cu⋯Cu distance in the dinuclear unit is 3.0225 (5) Å. There are previously reported dinuclear structures involving the ({[2-(pyridin-2-yl)eth­yl]imino)}meth­yl)phenolato ligand as a bridging ligand with other metals (Chakraborty *et al.*, 2013 ▸) and one instance involving copper (Yin *et al.*, 1998 ▸) where the structure is very similar apart from the fact that the bond between the Cu atom and the ClO_4_^−^ counter-ion is not indicated. There is very little information available for this structure apart from a line drawing in the Cambridge Structural Database (Groom *et al.*, 2016 ▸).

In the title structure (Fig. 1 ▸), since both Cu atoms are five-coordin­ate, the τ parameter (Addison *et al.*, 1984 ▸) for Cu1 is 0.21 while that for Cu2 is 0.045, indicating that Cu1 is more distorted from a square-pyramidal geometry than Cu2. The Cu—O bond lengths (Table 1 ▸) for Cu1 and Cu2 are 1.9469 (18), 2.0204 (17) Å and 1.9375 (18), 1.9545 (17) Å, respectively, while the Cu—N_imine_ and Cu—N_py_ bond lengths are 1.959 (2), 1.940 (2) Å and 1.996 (2), 1.987 (2) Å, respectively, with the bonds involving the imine group being shorter than those to pyridine as is generally found. The Cu1—OH_2_ and Cu2—OClO_3_ apical bonds are longer at 2.248 (2) and 2.6101 (18) Å, respectively.

The copper atoms are displaced from their basal coordination planes, O1, O2, N1, N2 (r.m.s. deviation = 0.186 Å) for Cu1, and O1, O2, N3, N4 (r.m.s. deviation = 0.252 Å) for Cu2, towards the apical ligands by 0.218 (1) and 0.037 (1) Å, respectively. The dihedral angle between these two planes is 39.31 (5)°. Thus the whole dinuclear complex adopts a saddle shape similar to that observed in metalloporphyrin structures (Kuzuhara *et al.*, 2016 ▸) with the two phenyl rings and two pyridine rings on opposite sides of the central Cu_2_O_2_ bridging group. The magnitude of this distortion can be seen from the dihedral angles between the two phenyl [41.45 (7)°] and the two pyridine rings [76.75 (7)°].

## Supra­molecular features   

**Figure 2:**
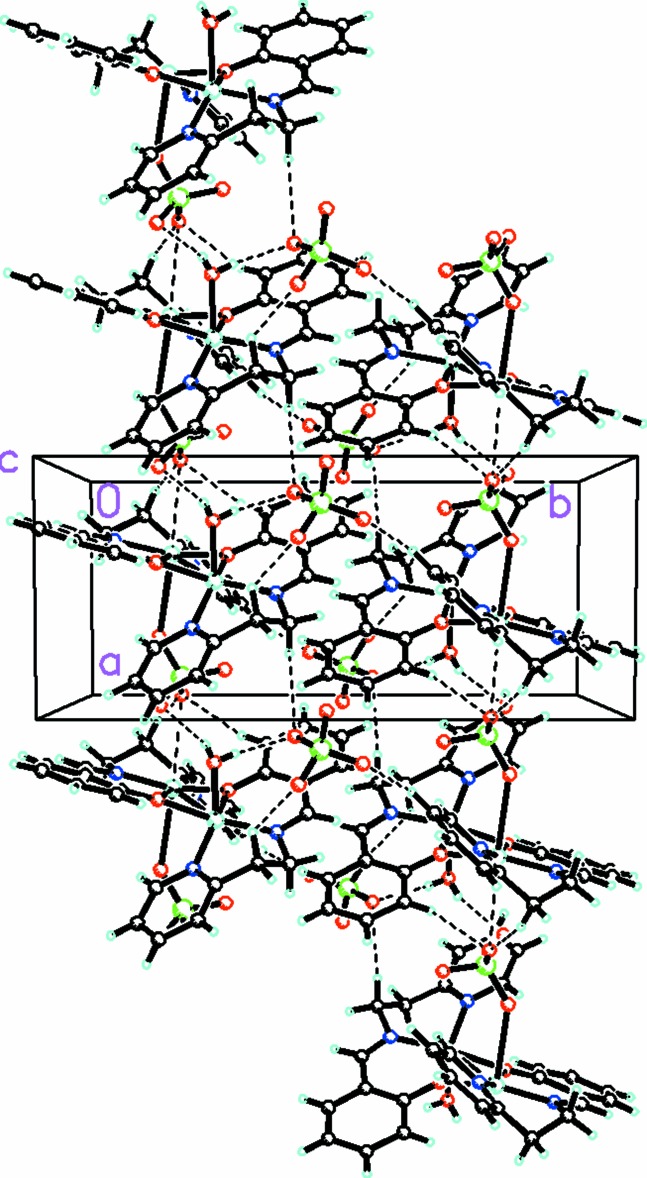
Packing diagram viewed along the *c* axis showing the extensive C—H⋯O and Cu⋯O inter­actions (dashed lines) linking the cations and anions into a complex three-dimensional array. Only the major occupancy conformations of the disordered anions are shown.

In addition to the bonds involving the copper atom mentioned above, there is a longer inter­action [2.9893 (5) Å] between Cu2 and O24 of an adjoining unit (at *x* + 1, y, *z*), which links the cations into linear chains along the *a-*axis direction (see Fig. 2 ▸). In addition, the water H atoms link with the perchlorate counter-ion. These inter­actions, along with numerous C—H⋯O inter­actions (Table 2 ▸) between the tetra­hedral perchlorate anions link into a complex three-dimensional array.

## Database survey   

A survey of the Cambridge Structural Database (Version 5.38; Groom *et al.*, 2016 ▸) for similar dinuclear structures of related Schiff base ligands and involving both coordinated perchlorate and water mol­ecules resulted in seven hits [COSHUO (Anbu *et al.*, 2009 ▸), EFUJAS (da Rocha *et al.*, 2014 ▸), EFUJEW (da Rocha *et al.*, 2014 ▸), JAVTOP (Mandal *et al.*, 1989 ▸), JAVTOP01 (Cheng *et al.*, 2012 ▸), WOGVAR (Cheng *et al.*, 2014 ▸), and WUKPAU (Hazra *et al.*, 2009 ▸)]. However, in all cases the ligands involved were tetra­dentate Schiff base macrocycles rather than tridentate Schiff base ligands. Thus there is no directly related example.

## Synthesis and crystallization   

2-(2-Pyrid­yl)ethyl­amine (0.3918 g, 3.207 mmol) was dissolved in methanol. Salicyl­aldehyde (0.3916 g, 3.207 mmol) was dissolved in methanol and stirred overnight. Cu(ClO_4_)_2_·6H_2_O (4.811 g, 1.783 mmol) was dissolved in the methanol solution. The mixture was stirred at room temperature overnight. The methanol was removed by rotary evaporation. The product was crystallized by dissolving it in aceto­nitrile and layering the solution with diethyl ether. The green crystals formed were allowed to grow overnight before gravity filtering, air drying, and collection of the crystallized product.

## Refinement   

Crystal data, data collection and structure refinement details are summarized in Table 3 ▸. The H atoms were positioned geometrically and allowed to ride on their parent atoms, with C—H = 0.95–0.99 Å and N—H = 1.00 Å and with *U*_iso_(H) = *xU*_eq_(C), where *x* = 1.5 for methyl H atoms and 1.2 for all other C-bound H atoms. The hydrogen atoms attached to water were refined isotropically. One of the perchlorate anions is disordered over two conformations with occupancies of 0.586 (4) and 0.414 (4) and were constrained to have similar thermal and metrical parameters.

## Supplementary Material

Crystal structure: contains datablock(s) I. DOI: 10.1107/S2056989017014694/hg5497sup1.cif

Structure factors: contains datablock(s) I. DOI: 10.1107/S2056989017014694/hg5497Isup2.hkl

CCDC reference: 1579206

Additional supporting information:  crystallographic information; 3D view; checkCIF report

## Figures and Tables

**Table 1:** Selected bond lengths (Å)

Cu1—O1	1.9469 (18)	Cu2—O2	1.9375 (18)
Cu1—N2	1.959 (2)	Cu2—N4	1.940 (2)
Cu1—N1	1.996 (2)	Cu2—O1	1.9545 (17)
Cu1—O2	2.0204 (17)	Cu2—N3	1.987 (2)
Cu1—O1*W*	2.248 (2)	Cu2—O21	2.6101 (18)
Cu1—Cu2	3.0225 (5)		

**Table 2:** Hydrogen-bond geometry (Å, °)

*D*—H⋯*A*	*D*—H	H⋯*A*	*D*⋯*A*	*D*—H⋯*A*
O1*W*—H1*W*1⋯O12	0.77 (4)	1.98 (4)	2.735 (4)	168 (4)
O1*W*—H1*W*1⋯O12*A*	0.77 (4)	2.06 (4)	2.769 (5)	153 (4)
O1*W*—H1*W*2⋯O23^i^	0.75 (4)	2.23 (4)	2.938 (4)	160 (4)
C2—H2*A*⋯N3	0.95	2.61	3.142 (3)	116
C2—H2*A*⋯O24^i^	0.95	2.55	3.196 (3)	125
C8—H8*A*⋯O12^ii^	0.99	2.54	3.488 (5)	161
C9—H9*A*⋯O13	0.99	2.40	3.121 (5)	129
C14—H14*A*⋯O2	0.95	2.54	3.073 (3)	116
C14—H14*A*⋯O21	0.95	2.60	3.345 (4)	135
C16—H16*A*⋯O1*W*	0.95	2.61	3.154 (4)	117
C16—H16*A*⋯N1	0.95	2.66	3.294 (3)	124
C23—H23*A*⋯O24^i^	0.99	2.44	3.336 (3)	151
C23—H23*B*⋯O13^iii^	0.99	2.55	3.293 (4)	132
C23—H23*B*⋯O13*A* ^iii^	0.99	2.54	3.261 (5)	129
C25—H25*A*⋯O13^iii^	0.95	2.55	3.175 (4)	124
C25—H25*A*⋯O12*A* ^iii^	0.95	2.58	3.488 (5)	159
C27—H27*A*⋯O14^iv^	0.95	2.39	3.202 (4)	143
C27—H27*A*⋯O14*A* ^iv^	0.95	2.62	3.477 (5)	151
C28—H28*A*⋯Cl2	0.95	2.99	3.594 (3)	123
C28—H28*A*⋯O22	0.95	2.61	3.473 (4)	151

Symmetry codes: (i) 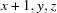; (ii) 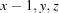; (iii) 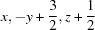; (iv) 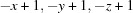.

**Table 3:** Experimental details

Crystal data	
Chemical formula	[Cu_2_(ClO_4_)(C_14_H_13_N_2_O)_2_(H_2_O)]ClO_4_
*M*_r_	794.52
Crystal system, space group	Monoclinic, *P*2_1_/*c*
Temperature (K)	100
*a*, *b*, *c* (Å)	7.4829 (4), 16.8867 (8), 24.2649 (13)
β (°)	98.180 (3)
*V* (Å^3^)	3035.0 (3)
*Z*	4
Radiation type	Mo *K*α
μ (mm^−1^)	1.65
Crystal size (mm)	0.33 × 0.27 × 0.09

Data collection	
Diffractometer	Bruker APEXII CCD
Absorption correction	Multi-scan (*SADABS*; Sheldrick, 1996 ▸)
*T*_min_, *T*_max_	0.616, 0.746
No. of measured, independent and observed [*I* > 2σ(*I*)] reflections	21194, 6730, 5328
*R*_int_	0.048
(sin θ/λ)_max_ (Å^−1^)	0.642

Refinement	
*R*[*F* ^2^ > 2σ(*F* ^2^)], *wR*(*F* ^2^), *S*	0.036, 0.084, 1.02
No. of reflections	6730
No. of parameters	470
No. of restraints	30
H-atom treatment	H atoms treated by a mixture of independent and constrained refinement
Δρ_max_, Δρ_min_ (e Å^−3^)	0.63, −0.52

Computer programs: *APEX3* and *SAINT* (Bruker, 2015 ▸), *SHELXT* (Sheldrick, 2015*a* ▸), *SHELXL2016* (Sheldrick, 2015*b* ▸) and *SHELXTL* (Sheldrick, 2008 ▸).

## supplementary crystallographic information

### Crystal data

**Table:**

[Cu_2_(ClO_4_)(C_14_H_13_N_2_O)_2_(H_2_O)]ClO_4_	*F*(000) = 1616
*M**_r_* = 794.52	*D*_x_ = 1.739 Mg m^−^^3^
Monoclinic, *P*2_1_/*c*	Mo *K*α radiation, λ = 0.71073 Å
*a* = 7.4829 (4) Å	Cell parameters from 4638 reflections
*b* = 16.8867 (8) Å	θ = 2.4–25.9°
*c* = 24.2649 (13) Å	µ = 1.65 mm^−^^1^
β = 98.180 (3)°	*T* = 100 K
*V* = 3035.0 (3) Å^3^	Plate, green
*Z* = 4	0.33 × 0.27 × 0.09 mm

### Data collection

**Table:**

Bruker APEXII CCD diffractometer	5328 reflections with *I* > 2σ(*I*)
ω and φ scans	*R*_int_ = 0.048
Absorption correction: multi-scan (SADABS; Sheldrick, 1996)	θ_max_ = 27.2°, θ_min_ = 1.5°
*T*_min_ = 0.616, *T*_max_ = 0.746	*h* = −9→9
21194 measured reflections	*k* = −21→21
6730 independent reflections	*l* = −26→31

### Refinement

**Table:**

Refinement on *F*^2^	30 restraints
Least-squares matrix: full	Hydrogen site location: mixed
*R*[*F*^2^ > 2σ(*F*^2^)] = 0.036	H atoms treated by a mixture of independent and constrained refinement
*wR*(*F*^2^) = 0.084	*w* = 1/[σ^2^(*F*_o_^2^) + (0.0319*P*)^2^ + 1.7524*P*] where *P* = (*F*_o_^2^ + 2*F*_c_^2^)/3
*S* = 1.02	(Δ/σ)_max_ = 0.001
6730 reflections	Δρ_max_ = 0.63 e Å^−^^3^
470 parameters	Δρ_min_ = −0.52 e Å^−^^3^

### Special details

**Table:**

Geometry. All esds (except the esd in the dihedral angle between two l.s. planes) are estimated using the full covariance matrix. The cell esds are taken into account individually in the estimation of esds in distances, angles and torsion angles; correlations between esds in cell parameters are only used when they are defined by crystal symmetry. An approximate (isotropic) treatment of cell esds is used for estimating esds involving l.s. planes.

### Fractional atomic coordinates and isotropic or equivalent isotropic displacement parameters (Å^2^)

**Table:**

	*x*	*y*	*z*	*U*_iso_*/*U*_eq_	Occ. (<1)
Cu1	0.53169 (4)	0.71966 (2)	0.40451 (2)	0.01326 (9)	
Cu2	0.64416 (4)	0.80469 (2)	0.51354 (2)	0.01310 (9)	
O1	0.6427 (2)	0.69899 (10)	0.48067 (7)	0.0151 (4)	
O2	0.6100 (2)	0.82773 (10)	0.43452 (7)	0.0135 (4)	
O1W	0.7788 (3)	0.71972 (15)	0.36159 (12)	0.0325 (6)	
H1W1	0.817 (5)	0.681 (2)	0.3507 (16)	0.039 (12)*	
H1W2	0.853 (6)	0.737 (2)	0.3817 (18)	0.052 (15)*	
N1	0.3377 (3)	0.75810 (14)	0.34554 (9)	0.0193 (5)	
N2	0.4813 (3)	0.60696 (13)	0.39084 (9)	0.0171 (5)	
N3	0.5957 (3)	0.76882 (12)	0.58799 (9)	0.0156 (5)	
N4	0.7050 (3)	0.91277 (12)	0.53591 (9)	0.0138 (5)	
C1	0.7146 (3)	0.63049 (15)	0.50207 (11)	0.0134 (5)	
C2	0.8316 (4)	0.62923 (16)	0.55200 (11)	0.0167 (6)	
H2A	0.863612	0.677602	0.570836	0.020*	
C3	0.9025 (4)	0.55874 (16)	0.57481 (12)	0.0205 (6)	
H3A	0.981089	0.559415	0.609192	0.025*	
C4	0.8600 (4)	0.48728 (16)	0.54803 (12)	0.0200 (6)	
H4A	0.907928	0.438987	0.563871	0.024*	
C5	0.7471 (4)	0.48759 (15)	0.49813 (12)	0.0193 (6)	
H5A	0.718640	0.438848	0.479376	0.023*	
C6	0.6727 (3)	0.55813 (15)	0.47409 (11)	0.0142 (5)	
C7	0.5537 (4)	0.55121 (16)	0.42186 (12)	0.0176 (6)	
H7A	0.526014	0.498850	0.409090	0.021*	
C8	0.3594 (4)	0.58258 (17)	0.34059 (12)	0.0244 (7)	
H8A	0.233446	0.582239	0.348750	0.029*	
H8B	0.390221	0.528104	0.330182	0.029*	
C9	0.3735 (4)	0.63824 (19)	0.29198 (12)	0.0289 (7)	
H9A	0.502428	0.647341	0.288949	0.035*	
H9B	0.316666	0.612975	0.257015	0.035*	
C10	0.2835 (4)	0.71639 (18)	0.29886 (12)	0.0249 (7)	
C11	0.1474 (5)	0.7452 (2)	0.25888 (14)	0.0369 (9)	
H11A	0.111041	0.715564	0.225904	0.044*	
C12	0.0651 (4)	0.8162 (2)	0.26675 (14)	0.0385 (9)	
H12A	−0.027315	0.836166	0.239380	0.046*	
C13	0.1191 (4)	0.8579 (2)	0.31509 (14)	0.0330 (8)	
H13A	0.063703	0.906905	0.321819	0.040*	
C14	0.2548 (4)	0.82716 (17)	0.35349 (12)	0.0227 (6)	
H14A	0.291391	0.855812	0.386882	0.027*	
C15	0.6555 (3)	0.89443 (15)	0.40998 (11)	0.0127 (5)	
C16	0.6481 (3)	0.89741 (16)	0.35177 (11)	0.0169 (6)	
H16A	0.613296	0.851503	0.330261	0.020*	
C17	0.6905 (4)	0.96585 (17)	0.32535 (12)	0.0213 (6)	
H17A	0.681549	0.967031	0.285911	0.026*	
C18	0.7463 (4)	1.03317 (17)	0.35622 (12)	0.0235 (6)	
H18A	0.776552	1.080168	0.338116	0.028*	
C19	0.7570 (4)	1.03079 (16)	0.41308 (12)	0.0205 (6)	
H19A	0.796181	1.076646	0.434061	0.025*	
C20	0.7118 (3)	0.96260 (15)	0.44124 (11)	0.0144 (5)	
C21	0.7319 (4)	0.96765 (15)	0.50133 (11)	0.0157 (6)	
H21A	0.769487	1.017465	0.517097	0.019*	
C22	0.7358 (4)	0.93284 (15)	0.59553 (11)	0.0175 (6)	
H22A	0.619579	0.947958	0.607637	0.021*	
H22B	0.817965	0.978951	0.601421	0.021*	
C23	0.8174 (4)	0.86374 (16)	0.63077 (11)	0.0171 (6)	
H23A	0.920277	0.842113	0.614010	0.020*	
H23B	0.864904	0.883347	0.668470	0.020*	
C24	0.6844 (4)	0.79844 (15)	0.63580 (11)	0.0165 (6)	
C25	0.6525 (4)	0.76883 (16)	0.68695 (11)	0.0215 (6)	
H25A	0.716010	0.789744	0.720479	0.026*	
C26	0.5278 (4)	0.70870 (16)	0.68878 (12)	0.0246 (7)	
H26A	0.506907	0.687092	0.723446	0.030*	
C27	0.4339 (4)	0.68053 (17)	0.63949 (12)	0.0246 (7)	
H27A	0.344882	0.640472	0.639727	0.030*	
C28	0.4720 (4)	0.71164 (16)	0.59010 (12)	0.0199 (6)	
H28A	0.408509	0.691964	0.556180	0.024*	
Cl1	0.84605 (9)	0.52555 (4)	0.28292 (3)	0.02048 (15)	
O11	1.0053 (5)	0.5149 (3)	0.25905 (18)	0.0327 (13)	0.586 (4)
O12	0.8916 (5)	0.57231 (19)	0.33492 (12)	0.0341 (11)	0.586 (4)
O13	0.7085 (5)	0.5647 (3)	0.24778 (15)	0.0414 (13)	0.586 (4)
O14	0.7827 (5)	0.45020 (19)	0.30101 (17)	0.0399 (12)	0.586 (4)
O11A	1.0101 (6)	0.4844 (3)	0.2771 (2)	0.0227 (15)	0.414 (4)
O12A	0.8864 (6)	0.60924 (18)	0.28866 (18)	0.0245 (14)	0.414 (4)
O13A	0.7265 (6)	0.5176 (3)	0.22912 (15)	0.0247 (13)	0.414 (4)
O14A	0.7602 (6)	0.4962 (3)	0.32548 (17)	0.0297 (15)	0.414 (4)
Cl2	0.12635 (9)	0.78538 (4)	0.48455 (3)	0.02373 (16)	
O21	0.2961 (2)	0.82746 (11)	0.49259 (8)	0.0234 (4)	
O22	0.1576 (3)	0.70495 (11)	0.47020 (10)	0.0387 (6)	
O23	0.0085 (3)	0.82239 (14)	0.43977 (10)	0.0504 (7)	
O24	0.0460 (3)	0.78881 (13)	0.53443 (10)	0.0478 (7)	

### Atomic displacement parameters (Å^2^)

**Table:**

	*U*^11^	*U*^22^	*U*^33^	*U*^12^	*U*^13^	*U*^23^
Cu1	0.01441 (17)	0.01313 (16)	0.01154 (16)	−0.00321 (12)	−0.00060 (12)	−0.00125 (13)
Cu2	0.01958 (18)	0.00892 (15)	0.01046 (16)	−0.00099 (13)	0.00097 (12)	−0.00080 (12)
O1	0.0207 (10)	0.0102 (9)	0.0131 (9)	0.0009 (7)	−0.0016 (8)	−0.0004 (7)
O2	0.0150 (9)	0.0123 (9)	0.0130 (9)	−0.0024 (7)	0.0014 (7)	−0.0006 (7)
O1W	0.0282 (14)	0.0264 (13)	0.0465 (16)	−0.0081 (11)	0.0175 (12)	−0.0170 (12)
N1	0.0192 (12)	0.0215 (12)	0.0161 (12)	−0.0064 (10)	−0.0012 (9)	0.0054 (10)
N2	0.0167 (12)	0.0193 (12)	0.0151 (12)	−0.0051 (9)	0.0015 (9)	−0.0066 (10)
N3	0.0208 (12)	0.0122 (11)	0.0140 (11)	0.0003 (9)	0.0036 (9)	−0.0008 (9)
N4	0.0155 (11)	0.0125 (11)	0.0128 (11)	0.0012 (9)	0.0004 (9)	−0.0025 (9)
C1	0.0126 (13)	0.0127 (13)	0.0150 (13)	−0.0008 (10)	0.0026 (10)	0.0014 (10)
C2	0.0183 (14)	0.0144 (13)	0.0172 (14)	−0.0016 (11)	0.0025 (11)	−0.0009 (11)
C3	0.0171 (14)	0.0192 (14)	0.0253 (16)	0.0008 (11)	0.0036 (12)	0.0076 (12)
C4	0.0169 (14)	0.0158 (14)	0.0289 (16)	0.0038 (11)	0.0086 (12)	0.0090 (12)
C5	0.0209 (15)	0.0095 (13)	0.0297 (16)	−0.0001 (11)	0.0108 (12)	−0.0006 (12)
C6	0.0121 (13)	0.0127 (13)	0.0184 (14)	−0.0019 (10)	0.0046 (10)	0.0012 (11)
C7	0.0177 (14)	0.0120 (13)	0.0240 (15)	−0.0045 (11)	0.0055 (12)	−0.0053 (11)
C8	0.0286 (17)	0.0223 (15)	0.0200 (15)	−0.0095 (13)	−0.0041 (12)	−0.0071 (12)
C9	0.0353 (18)	0.0369 (18)	0.0140 (14)	−0.0166 (15)	0.0017 (13)	−0.0082 (13)
C10	0.0237 (16)	0.0347 (17)	0.0146 (14)	−0.0182 (13)	−0.0026 (12)	0.0063 (13)
C11	0.036 (2)	0.048 (2)	0.0235 (17)	−0.0229 (17)	−0.0080 (14)	0.0092 (16)
C12	0.0251 (18)	0.053 (2)	0.0311 (19)	−0.0159 (16)	−0.0167 (14)	0.0215 (17)
C13	0.0207 (16)	0.0354 (18)	0.040 (2)	−0.0048 (14)	−0.0069 (14)	0.0178 (16)
C14	0.0196 (15)	0.0235 (15)	0.0240 (16)	−0.0066 (12)	−0.0010 (12)	0.0044 (13)
C15	0.0088 (12)	0.0137 (13)	0.0148 (13)	−0.0003 (10)	−0.0006 (10)	0.0031 (10)
C16	0.0153 (14)	0.0177 (14)	0.0169 (14)	−0.0034 (11)	−0.0004 (11)	0.0005 (11)
C17	0.0211 (15)	0.0279 (16)	0.0143 (14)	−0.0051 (12)	0.0003 (11)	0.0057 (12)
C18	0.0248 (16)	0.0210 (15)	0.0232 (16)	−0.0083 (12)	−0.0012 (12)	0.0104 (13)
C19	0.0232 (15)	0.0136 (13)	0.0238 (15)	−0.0038 (11)	0.0002 (12)	0.0015 (12)
C20	0.0135 (13)	0.0133 (13)	0.0160 (14)	0.0015 (10)	0.0005 (10)	0.0011 (11)
C21	0.0173 (14)	0.0083 (12)	0.0206 (14)	−0.0005 (10)	−0.0009 (11)	−0.0032 (11)
C22	0.0230 (15)	0.0138 (13)	0.0154 (14)	−0.0024 (11)	0.0019 (11)	−0.0031 (11)
C23	0.0194 (14)	0.0191 (14)	0.0124 (13)	−0.0025 (11)	0.0009 (11)	−0.0024 (11)
C24	0.0167 (14)	0.0158 (13)	0.0175 (14)	0.0051 (11)	0.0040 (11)	−0.0011 (11)
C25	0.0299 (16)	0.0223 (15)	0.0126 (13)	0.0070 (12)	0.0044 (12)	−0.0011 (12)
C26	0.0381 (18)	0.0204 (15)	0.0181 (15)	0.0036 (13)	0.0136 (13)	0.0034 (12)
C27	0.0319 (17)	0.0185 (15)	0.0259 (16)	−0.0025 (12)	0.0126 (13)	0.0004 (13)
C28	0.0255 (16)	0.0156 (14)	0.0188 (14)	−0.0007 (11)	0.0042 (12)	−0.0018 (11)
Cl1	0.0191 (3)	0.0175 (3)	0.0249 (4)	0.0010 (3)	0.0035 (3)	0.0016 (3)
O11	0.022 (2)	0.044 (3)	0.035 (3)	−0.008 (2)	0.014 (2)	−0.016 (2)
O12	0.051 (3)	0.027 (2)	0.023 (2)	0.0146 (18)	0.0019 (18)	−0.0074 (17)
O13	0.041 (3)	0.048 (3)	0.033 (3)	0.021 (2)	0.001 (2)	0.019 (2)
O14	0.058 (3)	0.031 (2)	0.032 (3)	−0.013 (2)	0.013 (2)	0.010 (2)
O11A	0.018 (3)	0.029 (4)	0.020 (3)	0.001 (2)	0.001 (2)	−0.008 (3)
O12A	0.036 (3)	0.012 (2)	0.026 (3)	−0.002 (2)	0.003 (2)	−0.003 (2)
O13A	0.025 (3)	0.024 (3)	0.021 (3)	−0.001 (2)	−0.007 (2)	−0.001 (2)
O14A	0.031 (3)	0.039 (4)	0.021 (3)	0.000 (3)	0.011 (2)	0.020 (3)
Cl2	0.0191 (4)	0.0171 (3)	0.0371 (4)	−0.0021 (3)	0.0110 (3)	−0.0043 (3)
O21	0.0161 (10)	0.0262 (11)	0.0285 (11)	−0.0041 (8)	0.0052 (8)	0.0001 (9)
O22	0.0504 (16)	0.0196 (11)	0.0480 (15)	−0.0036 (10)	0.0140 (12)	−0.0149 (11)
O23	0.0166 (12)	0.0536 (16)	0.077 (2)	0.0060 (11)	−0.0057 (12)	0.0130 (15)
O24	0.0596 (17)	0.0368 (14)	0.0570 (17)	−0.0252 (12)	0.0431 (14)	−0.0218 (12)

### Geometric parameters (Å, º)

**Table:**

Cu1—O1	1.9469 (18)	C12—C13	1.379 (5)
Cu1—N2	1.959 (2)	C12—H12A	0.9500
Cu1—N1	1.996 (2)	C13—C14	1.378 (4)
Cu1—O2	2.0204 (17)	C13—H13A	0.9500
Cu1—O1W	2.248 (2)	C14—H14A	0.9500
Cu1—Cu2	3.0225 (5)	C15—C16	1.407 (4)
Cu2—O2	1.9375 (18)	C15—C20	1.409 (4)
Cu2—N4	1.940 (2)	C16—C17	1.380 (4)
Cu2—O1	1.9545 (17)	C16—H16A	0.9500
Cu2—N3	1.987 (2)	C17—C18	1.392 (4)
Cu2—O21	2.6101 (18)	C17—H17A	0.9500
O1—C1	1.348 (3)	C18—C19	1.371 (4)
O2—C15	1.340 (3)	C18—H18A	0.9500
O1W—H1W1	0.77 (4)	C19—C20	1.405 (4)
O1W—H1W2	0.75 (4)	C19—H19A	0.9500
N1—C10	1.347 (4)	C20—C21	1.447 (4)
N1—C14	1.348 (4)	C21—H21A	0.9500
N2—C7	1.277 (3)	C22—C23	1.522 (4)
N2—C8	1.474 (3)	C22—H22A	0.9900
N3—C28	1.343 (3)	C22—H22B	0.9900
N3—C24	1.348 (3)	C23—C24	1.502 (4)
N4—C21	1.285 (3)	C23—H23A	0.9900
N4—C22	1.472 (3)	C23—H23B	0.9900
C1—C2	1.390 (4)	C24—C25	1.390 (4)
C1—C6	1.411 (4)	C25—C26	1.384 (4)
C2—C3	1.386 (4)	C25—H25A	0.9500
C2—H2A	0.9500	C26—C27	1.382 (4)
C3—C4	1.386 (4)	C26—H26A	0.9500
C3—H3A	0.9500	C27—C28	1.375 (4)
C4—C5	1.374 (4)	C27—H27A	0.9500
C4—H4A	0.9500	C28—H28A	0.9500
C5—C6	1.406 (4)	Cl1—O14A	1.383 (3)
C5—H5A	0.9500	Cl1—O13	1.405 (3)
C6—C7	1.446 (4)	Cl1—O11	1.408 (3)
C7—H7A	0.9500	Cl1—O11A	1.435 (3)
C8—C9	1.524 (4)	Cl1—O14	1.448 (3)
C8—H8A	0.9900	Cl1—O12A	1.448 (3)
C8—H8B	0.9900	Cl1—O13A	1.480 (3)
C9—C10	1.502 (4)	Cl1—O12	1.486 (3)
C9—H9A	0.9900	Cl2—O24	1.4271 (19)
C9—H9B	0.9900	Cl2—O22	1.4296 (18)
C10—C11	1.391 (4)	Cl2—O23	1.441 (2)
C11—C12	1.373 (5)	Cl2—O21	1.4443 (18)
C11—H11A	0.9500		
			
O1—Cu1—N2	91.85 (8)	H9A—C9—H9B	107.9
O1—Cu1—N1	155.14 (9)	N1—C10—C11	120.5 (3)
N2—Cu1—N1	95.26 (10)	N1—C10—C9	117.8 (3)
O1—Cu1—O2	75.95 (7)	C11—C10—C9	121.7 (3)
N2—Cu1—O2	167.78 (8)	C12—C11—C10	120.4 (3)
N1—Cu1—O2	96.23 (8)	C12—C11—H11A	119.8
O1—Cu1—O1W	99.89 (9)	C10—C11—H11A	119.8
N2—Cu1—O1W	94.19 (9)	C11—C12—C13	118.8 (3)
N1—Cu1—O1W	103.30 (10)	C11—C12—H12A	120.6
O2—Cu1—O1W	87.20 (9)	C13—C12—H12A	120.6
O1—Cu1—Cu2	39.31 (5)	C14—C13—C12	118.8 (3)
N2—Cu1—Cu2	129.24 (7)	C14—C13—H13A	120.6
N1—Cu1—Cu2	123.81 (7)	C12—C13—H13A	120.6
O2—Cu1—Cu2	39.21 (5)	N1—C14—C13	122.6 (3)
O1W—Cu1—Cu2	105.09 (8)	N1—C14—H14A	118.7
O2—Cu2—N4	94.63 (8)	C13—C14—H14A	118.7
O2—Cu2—O1	77.71 (7)	O2—C15—C16	120.0 (2)
N4—Cu2—O1	163.92 (8)	O2—C15—C20	121.5 (2)
O2—Cu2—N3	161.00 (9)	C16—C15—C20	118.5 (2)
N4—Cu2—N3	95.65 (9)	C17—C16—C15	121.2 (3)
O1—Cu2—N3	95.83 (8)	C17—C16—H16A	119.4
O2—Cu2—O21	77.88 (7)	C15—C16—H16A	119.4
N4—Cu2—O21	95.97 (8)	C16—C17—C18	120.3 (3)
O1—Cu2—O21	96.18 (7)	C16—C17—H17A	119.8
N3—Cu2—O21	85.18 (8)	C18—C17—H17A	119.8
O2—Cu2—Cu1	41.24 (5)	C19—C18—C17	119.2 (3)
N4—Cu2—Cu1	135.80 (7)	C19—C18—H18A	120.4
O1—Cu2—Cu1	39.13 (5)	C17—C18—H18A	120.4
N3—Cu2—Cu1	125.94 (6)	C18—C19—C20	122.0 (3)
O21—Cu2—Cu1	75.74 (4)	C18—C19—H19A	119.0
C1—O1—Cu1	127.77 (16)	C20—C19—H19A	119.0
C1—O1—Cu2	130.37 (16)	C19—C20—C15	118.8 (2)
Cu1—O1—Cu2	101.56 (8)	C19—C20—C21	116.4 (2)
C15—O2—Cu2	127.02 (16)	C15—C20—C21	124.8 (2)
C15—O2—Cu1	132.68 (16)	N4—C21—C20	127.7 (2)
Cu2—O2—Cu1	99.55 (8)	N4—C21—H21A	116.1
Cu1—O1W—H1W1	122 (3)	C20—C21—H21A	116.1
Cu1—O1W—H1W2	106 (3)	N4—C22—C23	111.7 (2)
H1W1—O1W—H1W2	106 (4)	N4—C22—H22A	109.3
C10—N1—C14	118.9 (3)	C23—C22—H22A	109.3
C10—N1—Cu1	122.3 (2)	N4—C22—H22B	109.3
C14—N1—Cu1	118.78 (19)	C23—C22—H22B	109.3
C7—N2—C8	116.3 (2)	H22A—C22—H22B	107.9
C7—N2—Cu1	124.14 (19)	C24—C23—C22	113.0 (2)
C8—N2—Cu1	119.54 (18)	C24—C23—H23A	109.0
C28—N3—C24	119.4 (2)	C22—C23—H23A	109.0
C28—N3—Cu2	118.00 (18)	C24—C23—H23B	109.0
C24—N3—Cu2	122.56 (18)	C22—C23—H23B	109.0
C21—N4—C22	117.3 (2)	H23A—C23—H23B	107.8
C21—N4—Cu2	123.24 (18)	N3—C24—C25	120.6 (3)
C22—N4—Cu2	119.30 (16)	N3—C24—C23	117.0 (2)
O1—C1—C2	121.1 (2)	C25—C24—C23	122.5 (3)
O1—C1—C6	120.6 (2)	C26—C25—C24	119.7 (3)
C2—C1—C6	118.4 (2)	C26—C25—H25A	120.2
C3—C2—C1	121.3 (3)	C24—C25—H25A	120.2
C3—C2—H2A	119.4	C27—C26—C25	119.1 (3)
C1—C2—H2A	119.4	C27—C26—H26A	120.4
C2—C3—C4	120.7 (3)	C25—C26—H26A	120.4
C2—C3—H3A	119.6	C28—C27—C26	118.7 (3)
C4—C3—H3A	119.6	C28—C27—H27A	120.7
C5—C4—C3	118.8 (3)	C26—C27—H27A	120.7
C5—C4—H4A	120.6	N3—C28—C27	122.5 (3)
C3—C4—H4A	120.6	N3—C28—H28A	118.8
C4—C5—C6	121.8 (3)	C27—C28—H28A	118.8
C4—C5—H5A	119.1	O13—Cl1—O11	113.6 (2)
C6—C5—H5A	119.1	O14A—Cl1—O11A	113.2 (3)
C5—C6—C1	119.1 (2)	O13—Cl1—O14	110.8 (2)
C5—C6—C7	117.0 (2)	O11—Cl1—O14	110.2 (2)
C1—C6—C7	123.9 (2)	O14A—Cl1—O12A	113.0 (3)
N2—C7—C6	127.9 (2)	O11A—Cl1—O12A	108.2 (3)
N2—C7—H7A	116.1	O14A—Cl1—O13A	109.8 (2)
C6—C7—H7A	116.1	O11A—Cl1—O13A	106.7 (3)
N2—C8—C9	111.4 (2)	O12A—Cl1—O13A	105.4 (2)
N2—C8—H8A	109.3	O13—Cl1—O12	108.9 (2)
C9—C8—H8A	109.3	O11—Cl1—O12	108.2 (2)
N2—C8—H8B	109.3	O14—Cl1—O12	104.8 (2)
C9—C8—H8B	109.3	O24—Cl2—O22	110.39 (13)
H8A—C8—H8B	108.0	O24—Cl2—O23	109.59 (15)
C10—C9—C8	112.0 (2)	O22—Cl2—O23	109.41 (14)
C10—C9—H9A	109.2	O24—Cl2—O21	109.51 (13)
C8—C9—H9A	109.2	O22—Cl2—O21	109.20 (12)
C10—C9—H9B	109.2	O23—Cl2—O21	108.70 (13)
C8—C9—H9B	109.2	Cl2—O21—Cu2	141.99 (12)
			
Cu1—O1—C1—C2	162.18 (18)	Cu2—O2—C15—C20	−10.0 (3)
Cu2—O1—C1—C2	−10.3 (3)	Cu1—O2—C15—C20	−177.95 (17)
Cu1—O1—C1—C6	−17.7 (3)	O2—C15—C16—C17	178.6 (2)
Cu2—O1—C1—C6	169.79 (17)	C20—C15—C16—C17	−1.5 (4)
O1—C1—C2—C3	178.5 (2)	C15—C16—C17—C18	1.7 (4)
C6—C1—C2—C3	−1.7 (4)	C16—C17—C18—C19	−0.6 (4)
C1—C2—C3—C4	0.7 (4)	C17—C18—C19—C20	−0.6 (4)
C2—C3—C4—C5	0.5 (4)	C18—C19—C20—C15	0.7 (4)
C3—C4—C5—C6	−0.7 (4)	C18—C19—C20—C21	179.0 (3)
C4—C5—C6—C1	−0.2 (4)	O2—C15—C20—C19	−179.7 (2)
C4—C5—C6—C7	−179.1 (2)	C16—C15—C20—C19	0.4 (4)
O1—C1—C6—C5	−178.7 (2)	O2—C15—C20—C21	2.1 (4)
C2—C1—C6—C5	1.4 (4)	C16—C15—C20—C21	−177.8 (2)
O1—C1—C6—C7	0.1 (4)	C22—N4—C21—C20	179.8 (2)
C2—C1—C6—C7	−179.8 (2)	Cu2—N4—C21—C20	4.4 (4)
C8—N2—C7—C6	−177.8 (3)	C19—C20—C21—N4	−177.5 (3)
Cu1—N2—C7—C6	5.1 (4)	C15—C20—C21—N4	0.8 (4)
C5—C6—C7—N2	−174.8 (3)	C21—N4—C22—C23	−143.3 (2)
C1—C6—C7—N2	6.4 (4)	Cu2—N4—C22—C23	32.2 (3)
C7—N2—C8—C9	−144.5 (3)	N4—C22—C23—C24	−73.3 (3)
Cu1—N2—C8—C9	32.8 (3)	C28—N3—C24—C25	1.8 (4)
N2—C8—C9—C10	−73.9 (3)	Cu2—N3—C24—C25	−176.81 (19)
C14—N1—C10—C11	1.7 (4)	C28—N3—C24—C23	−178.4 (2)
Cu1—N1—C10—C11	179.8 (2)	Cu2—N3—C24—C23	3.0 (3)
C14—N1—C10—C9	−177.8 (2)	C22—C23—C24—N3	53.7 (3)
Cu1—N1—C10—C9	0.3 (3)	C22—C23—C24—C25	−126.4 (3)
C8—C9—C10—N1	56.1 (3)	N3—C24—C25—C26	−0.4 (4)
C8—C9—C10—C11	−123.4 (3)	C23—C24—C25—C26	179.8 (3)
N1—C10—C11—C12	−0.7 (4)	C24—C25—C26—C27	−1.5 (4)
C9—C10—C11—C12	178.8 (3)	C25—C26—C27—C28	2.0 (4)
C10—C11—C12—C13	−0.5 (5)	C24—N3—C28—C27	−1.3 (4)
C11—C12—C13—C14	0.6 (5)	Cu2—N3—C28—C27	177.4 (2)
C10—N1—C14—C13	−1.5 (4)	C26—C27—C28—N3	−0.6 (4)
Cu1—N1—C14—C13	−179.7 (2)	O24—Cl2—O21—Cu2	100.6 (2)
C12—C13—C14—N1	0.4 (5)	O22—Cl2—O21—Cu2	−20.4 (2)
Cu2—O2—C15—C16	169.93 (18)	O23—Cl2—O21—Cu2	−139.70 (19)
Cu1—O2—C15—C16	1.9 (4)		

### Hydrogen-bond geometry (Å, º)

**Table:**

*D*—H···*A*	*D*—H	H···*A*	*D*···*A*	*D*—H···*A*
O1*W*—H1*W*1···O12	0.77 (4)	1.98 (4)	2.735 (4)	168 (4)
O1*W*—H1*W*1···O12*A*	0.77 (4)	2.06 (4)	2.769 (5)	153 (4)
O1*W*—H1*W*2···O23^i^	0.75 (4)	2.23 (4)	2.938 (4)	160 (4)
C2—H2*A*···N3	0.95	2.61	3.142 (3)	116
C2—H2*A*···O24^i^	0.95	2.55	3.196 (3)	125
C8—H8*A*···O12^ii^	0.99	2.54	3.488 (5)	161
C9—H9*A*···O13	0.99	2.40	3.121 (5)	129
C14—H14*A*···O2	0.95	2.54	3.073 (3)	116
C14—H14*A*···O21	0.95	2.60	3.345 (4)	135
C16—H16*A*···O1*W*	0.95	2.61	3.154 (4)	117
C16—H16*A*···N1	0.95	2.66	3.294 (3)	124
C23—H23*A*···O24^i^	0.99	2.44	3.336 (3)	151
C23—H23*B*···O13^iii^	0.99	2.55	3.293 (4)	132
C23—H23*B*···O13*A*^iii^	0.99	2.54	3.261 (5)	129
C25—H25*A*···O13^iii^	0.95	2.55	3.175 (4)	124
C25—H25*A*···O12*A*^iii^	0.95	2.58	3.488 (5)	159
C27—H27*A*···O14^iv^	0.95	2.39	3.202 (4)	143
C27—H27*A*···O14*A*^iv^	0.95	2.62	3.477 (5)	151
C28—H28*A*···Cl2	0.95	2.99	3.594 (3)	123
C28—H28*A*···O22	0.95	2.61	3.473 (4)	151

Symmetry codes: (i) *x*+1, *y*, *z*; (ii) *x*−1, *y*, *z*; (iii) *x*, −*y*+3/2, *z*+1/2; (iv) −*x*+1, −*y*+1, −*z*+1.
